# Presumptive Bilirubin-Related Chlorodontia and Developmental Enamel Defects of the Primary Dentition in an Extremely Preterm Infant: A Case Report

**DOI:** 10.3390/jcm15145423

**Published:** 2026-07-10

**Authors:** Michalina Szymczak-Paluch, Agnieszka Bruzda-Zwiech, Sebastian Kłosek

**Affiliations:** 1 Department of Oral Pathology, Medical University of Lodz, Pomorska 251, 92-213 Lodz, Poland; sebastian.klosek@umed.lodz.pl; 2 Department of Developmental Age Dentistry, Medical University of Lodz, Pomorska 251, 92-213 Lodz, Poland; agnieszka.bruzda-zwiech@umed.lodz.pl

**Keywords:** chlorodontia, green teeth, neonatal hyperbilirubinemia, cholestasis, prematurity, enamel developmental defects, primary teeth

## Abstract

**Background**: Chlorodontia is a rare condition characterized by intrinsic green discoloration of teeth. It is most often reported in association with bilirubin pigment deposition during teeth development, in cases of severe and/or prolonged neonatal hyperbilirubinemia. In extremely preterm infants, this condition may be complicated by other developmental enamel defects (DDE), such as hypoplasia or hypomineralization, linked to prematurity, systemic diseases, nutritional disturbances, and intensive care exposures. That overlap of enamel abnormalities can make diagnosis more difficult, necessitate a complex treatment plan, and increase the unpredictability of dental treatment efficacy. **Case Presentation**: This report presents the case of a child born at 25 weeks’ gestation with a birth weight of 910 g. Her neonatal course was complicated by, among others, recurrent episodes of hyperbilirubinemia, first neonatal (treated with phototherapy between the second and fifth day of life), and afterwards due to cholestasis from day 30 of life, with coexisting bacterial sepsis and metabolic disturbances. The available neonatal medical documentation indicated that total bilirubin level peak took place on the 40th day of life with levels approaching approximately 30 mg/dL, as well as levels of conjugated bilirubin being 19.0 mg/dL, but the exact peak values, duration, and bilirubin fractionation were not given in the patient’s discharge form. At 15 months of chronological age (11.5 months corrected age), she was referred for an assessment of abnormal morphology and green discoloration of the erupted primary incisors. Clinical examination revealed intrinsic green discoloration of the teeth, rough incisal edges and enamel breakdown on the incisal third. During follow-up, less intensity of the green pigmentation in the subsequent groups of erupted teeth was noticed. Despite excellent oral hygiene and adherence to a low-cariogenic diet, the primary first molars probably developed post-eruptive enamel loss with exposed dentin tissue. Minimally invasive management was introduced using atraumatic restorative treatment with glass-ionomer cement, combined with intensive preventive care. **Conclusions**: Despite the fact that, in the presented case, the diagnosis of presumptive bilirubin-related green pigmentation relies exclusively on the clinical picture and the complex neonatal medical history, it shows that, in extremely preterm infants, chlorodontia may coexist with hypomineralization or hypoplasia. This requires the introduction of dental treatment and prophylaxis, adjusted to the child’s age, to lower the risk of further complications of DDEs.

## 1. Introduction

Chlorodontia is a very rare type of intrinsic dental discoloration which presents with green, yellow-greenish, or dark green pigmentation within the hard tissues of a tooth or a group of teeth. Unlike extrinsic staining—which may appear on a tooth surface and can be removed mechanically—intrinsic discolorations are a result of other pigments being incorporated into enamel and/or dentin tissues during odontogenesis. In pediatric populations, green stains found in the primary dentition has been reported most often in association with severe or prolonged neonatal hyperbilirubinemia and cholestatic jaundice, including cases linked to sepsis and prematurity [1,2,3,4]. Bilirubin is extensively deposited throughout the body during hyperbilirubinemia, including its incorporation into enamel and dentin during mineralization and crown formation. Once entrapped, this pigment persists, because mature enamel and dentin have no meaningful metabolic turnover, unlike soft tissues. As a result, the discoloration of teeth may only become apparent after eruption, functioning as a delayed oral indicator of significant systemic disturbance during the neonatal period [1,2,3].

Preterm infants, especially extremely preterm ones, are exceptionally susceptible to systemic conditions that can disrupt both bilirubin metabolism and dental hard-tissue development. Hepatic immaturity, cholestasis, infection/sepsis, prolonged intensive care, respiratory conditions, metabolic instability, and multiple medications that are often used coincide within key windows of enamel and dentin formation [5,6]. Prematurity and very low birth weight are also associated with higher rates of developmental defects of enamel (DDE) in the primary dentition, including enamel hypoplasia and qualitative mineralization abnormalities [7,8]. More recent clinical evidence suggests that the neonatal respiratory morbidity and related interventions may further increase the severity of these enamel defects in very preterm children [9,10].

Although neonatal jaundice is common, even among full-term newborns with normal birth weight, chlorodontia remains rare. Reports describing bilirubin-related green pigmentation occurring alongside severe developmental mineralization defects and post-eruptive enamel breakdown are particularly scarce. For this reason, the present case report describes the coexistence of presumptive bilirubin-associated chlorodontia DDEs in the primary dentition of an extremely preterm female infant with a complex neonatal course. It particularly aims to present the clinical appearance of the teeth, the diagnostic considerations and the preventive and minimally invasive restorative management of the defects of the hard dental tissues, that can be introduced in young children (under three years of age) to reduce the risk of DDE complications. However, the presented case report only outlines probable prenatal and postnatal factors, rather than constituting proof of underlying mechanisms.

## 2. Materials and Methods

This manuscript is considered as a single-patient clinical case report as it is based on a retrospective review of the patient’s medical history, dental records, clinical examinations, and photographic documentation. The report was structured according to MDPI case-report guidance, and the 2013 CARE checklist (required by a publisher) has been provided as an additional file [11].

The patient was examined and treated by an experienced pediatric dentistry specialist and a dentist undergoing her last year of residency training in this field, at the Pediatric Dentistry Outpatient Clinic of the Central Clinical Hospital of the Medical University of Lodz. Dental assessments were conducted during consecutive visits at chronological ages of 15, 18, and 20 months; corrected age was applied due to the patient’s extreme prematurity.

Because this was a single descriptive case report and not an epidemiological study, formal examiner calibration was not undertaken. This limitation is acknowledged below. However, dental examination of the patient was carried out in the presence of both dentists and the diagnosis was established collectively.

Written informed consent for the treatment and the publication of a paper that consists of anonymized intraoral clinical images, as well as anonymized medical and personal data, was obtained from the legal guardian. Under the applicable local rules for anonymized single-patient case reports, separate institutional review board approval was not required.

The medical review contained prenatal and perinatal history, gestational age, birth weight, Apgar scores, neonatal complications, systemic diagnoses, bilirubin-related disorders, neonatal intensive care course, surgical interventions, infections, respiratory complications, endocrine and metabolic abnormalities, medication exposure, and current specialist follow-up. Neonatal medical records were reviewed to establish the timing and duration of bilirubin elevation, bilirubin fractionation, duration of cholestasis, phototherapy details, exposure to intensive care and medication, and systemic complications that overlapped with tooth development. As these data were based solely on Patient Discharge Forms, any unavailable medical data are explicitly identified in the case description and the limitations section.

Dental evaluation included an extraoral and intraoral examination, documentation of erupted primary teeth using FDI notation system, assessment of eruption sequence, tooth color, tooth position, enamel morphology, post-eruptive enamel breakdown, dentin exposure, caries status, oral hygiene status, and mucosal health, as well as planning preventive measures, restorative treatment, and follow-up recommendations.

Teeth staining was described qualitatively, focusing on its location and color and its intensity. The enamel defects were classified based on their macroscopic appearance according to modified DDE Index for use in screening surveys (mDDE), which includes diffuse opacities, demarcated opacities, enamel hypoplasia, and other defects (e.g., stainings or combination of more than one type of defects) [12].

DDEs extent was assessed as being less than 1/3, at least 1/3 but less than 2/3, and at least 2/3. The presence of opacities and/or post-eruptive enamel breakdown was interpreted as hypomineralisation, whereas defects associated with a reduced localized thickness of enamel were interpreted as hypoplasia [7,12]. Differentiation between hypoplasia and hypomineralisation were based on the EAPD recommendations [13]. According to the EAPD recommendations for hypoplasia, the borders of normal enamel are mostly regular and smooth. In contrast, in cases of post-eruptive enamel substance loss, the enamel edges are sharp and irregular where the enamel has chipped off.

Early childhood caries was assessed clinically by visual inspection under the level of cooperation achievable at the visit. Caries was considered unlikely to be the initiating process since the defects appeared shortly after eruption on structurally abnormal occlusal surfaces, oral hygiene was excellent (OHI = 0, API = 0), the diet was low cariogenic, and no plaque-associated cavitated lesions or other clinical signs of active caries were observed. Radiographs were not obtained due to the patient’s young age, limited cooperation, and lack of urgent necessity for radiographic assessment.

The diagnosis was established based on macroscopic appearance of intrinsic green discoloration of erupted primary teeth and developmental defects, as well as a history of recurrent neonatal hyperbilirubinemia and cholestasis. No histopathological confirmation was obtained since extraction of affected teeth was not clinically indicated, nor did exfoliation occur during the follow-up period. The differential diagnosis included extrinsic discolorations, caused due to chromogenic bacterial deposits, dietary staining, medication-related discoloration, genetic enamel disorders, early childhood caries, and developmental enamel defects unrelated to bilirubin exposure.

## 3. Case Presentation

A female infant aged 15 months, corresponding to a corrected age of 11.5 months, was referred with her parents to the Pediatric Dentistry Outpatient Clinic of the Central Clinical Hospital of the Medical University of Lodz for diagnosis and treatment of primary teeth with abnormal morphology and unusual green discoloration. The child had not previously undergone any dental examination.

### 3.1. Prenatal, Perinatal, and Neonatal History

The patient was born by cesarean section at 25 weeks of gestation because of a life-threatening fetal condition. No maternal comorbidities, tobacco smoking, or use of prohibited substances during pregnancy were reported.

The patient was born extremely preterm with a birth weight of 910 g. The Apgar scores were 6, 6, 7, and 7 points. Her neonatal course was complicated by, among others, recurrent episodes of hyperbilirubinemia, first neonatal (treated with phototherapy between the second and fifth day of life), and afterwards due to cholestasis from day 30 of life, with coexisting bacterial sepsis (associated with *Staphylococcus haemolyticus*, *Klepsiella pneumoniae*, *Stenotrophomonas maltophilia*) and metabolic disturbances. The available neonatal medical documentation indicated that extremal hyperbilirubinemia with total bilirubin level peak took place on the 40th day of life (bilirubin levels approaching approximately 30 mg/dL, as well as levels of conjugated bilirubin being 19.0 mg/dL), but exact peak values, duration, and bilirubin fractionation were not given in the patient’s discharge form.

The available records did not specify the duration of cholestasis, nor did they provide detailed phototherapy parameters (including total duration, intensity, or number of sessions). They also did not allow complete chronological reconstruction of neonatal medication exposure or NICU interventions during odontogenesis.

Three months before the first dental visit, the child was hospitalized in a pediatric department because of interstitial pneumonia and respiratory tract infection, in the course of which the child was investigated for primary immunodeficiency. During neonatal hospitalization, the patient received broad antimicrobial, antifungal, respiratory, cardiovascular, neurological, endocrine, and supportive treatment. The recorded medical regiments and dietary supplements, together with their corresponding international names or active substances, are summarized below (Table 1). Based on the available medical documentation, none of the substances recorded during neonatal hospitalization appears to be a recognized cause of green intrinsic tooth discoloration. In addition, tetracyclines which are known to adversely affect tooth development and cause staining were not administered to the mother during pregnancy or breastfeeding. At the time of dental evaluations, the patient remained under multidisciplinary specialist care, including pediatric, ophthalmological, neurological, endocrinological, cardiological, and physiotherapeutic follow-up.

### 3.2. Dental and Dietary History

The parents reported brushing the child’s teeth twice a day with fluoride toothpaste containing 1000 ppm fluoride. The amount of toothpaste used was consistent with the recommended rice-grain quantity for this age. The diet was assessed as having low cariogenic potential.

The parents were highly engaged in daily care, and oral hygiene was very good.

The main parents’ complaint was the severe green teeth discoloration of their child’s teeth and the enamel chipping off.

No history of enamel abnormalities that run in the family was reported.

### 3.3. Extraoral Examination

Extraoral examination revealed facial symmetry. Submental and submandibular lymph nodes were not palpable. Temporomandibular joint mobility was normal, with a normal mandibular opening pathway and no acoustic symptoms. Reduced muscle tone was observed. The child tended to thrust the tongue between the lips. The lips were hypotonic, and marked salivation was present.

### 3.4. First Dental Visit—15 Months Chronological Age, 11.5 Months Corrected Age

During the first visit, the child was 15 months old chronologically and 11.5 months old according to corrected age. Cooperation was limited because of the patient’s age.

**Figure 1:**
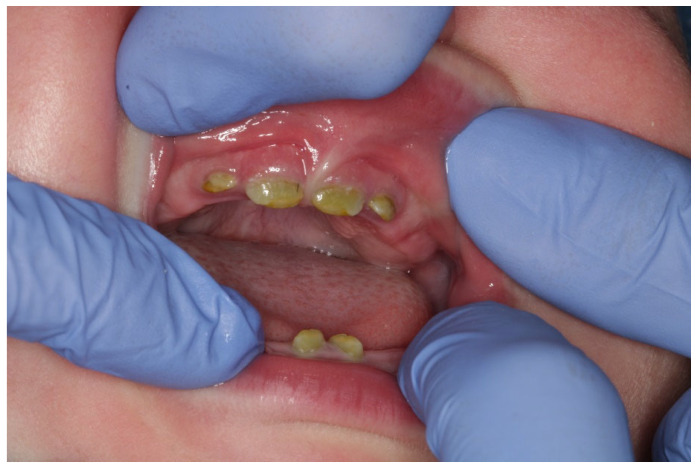
Green discoloration of the crowns of erupted primary teeth in the maxillary and mandibular arches in an extremely preterm infant with a history of neonatal cholestasis and recurrent hyperbilirubinemia. Developmental mineralization defects are visible. The mandibular central incisors are erupting in mesial rotation. The visible findings include intrinsic discoloration, irregular incisal morphology, and enamel loss probably due to post-eruptive breakdown.

The following primary teeth were present: 52, 51, 61, 62, 71, and 81. Because of the corrected age, delayed eruption was not diagnosed at this stage. All erupted primary teeth showed generalized intrinsic green discoloration affecting the entire teeth crowns. The incisal edges of teeth 51 and 61 were rough and loss of enamel involving 1/3 to 2/3 of labial surfaces of tooth 61 and up to 1/3 of teeth 51 and 52 were detected (Figure 1). The defects could be classified according to m-DDE Index as other defects due to presence of staining and a combination of more than one type of defects. The teeth 71 and 81 were erupting in mesial rotation (Table 2). The oral mucosa showed no clinical abnormalities. The labial and lingual frenula had normal morphology.

Preventive treatment was performed using fluoride varnish containing 5% sodium fluoride. The parents were instructed to continue brushing their child’s teeth twice daily using a rice-grain sized amount of 1000 ppm fluoride toothpaste. Home use of casein phosphopeptide-amorphous calcium phosphate cream was recommended. A follow-up visit was scheduled after three months.

### 3.5. Second Dental Visit—18 Months Chronological Age, 14.5 Months Corrected Age

During the second visit, the child was 18 months old chronologically and 14.5 months old according to corrected age. Cooperation remained limited because of the patient’s age.

**Figure 2:**
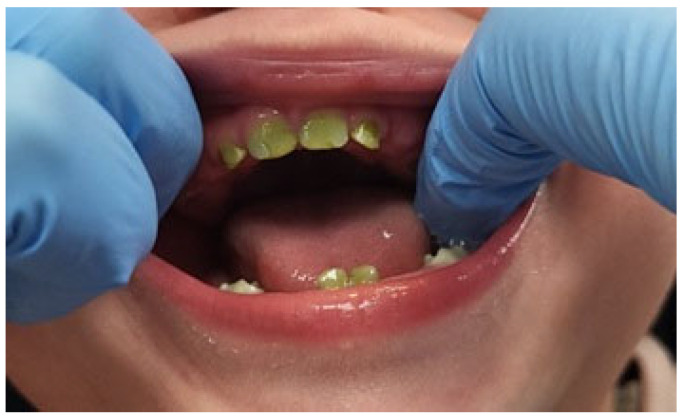
Generalized green discoloration of primary incisors and molars in an extremely preterm infant with a history of neonatal cholestasis. Developmental mineralization defects affect both anterior and posterior primary teeth, but first lower molars are discolored to a lesser degree than incisors.

The following teeth were present: 52, 51, 61, 62, 71, 74, 81, and 84. The cusps of erupting teeth 54 and 64 were visible in oral cavity. Teeth 74 and 84 were fully erupted. The gingivae of the alveolar ridge between teeth 71 and 74 and between teeth 81 and 84 were smooth, with no visible signs of eruption of the mandibular lateral incisors in these regions (Figure 2). The eruption sequence in the mandible was therefore irregular, with lack of clinical signs of eruption of the mandibular primary lateral incisors. Oral hygiene was excellent, with OHI = 0 and API = 0.

On the occlusal surfaces of teeth 74 and 84, enamel loss was observed, affecting more than 2/3 of these surfaces. Exposed dentin was soft. No clinical signs of caries were noted. Clinical picture suggested post-eruptive breakdown of structurally weakened enamel rather than carious destruction.

**Figure 3:**
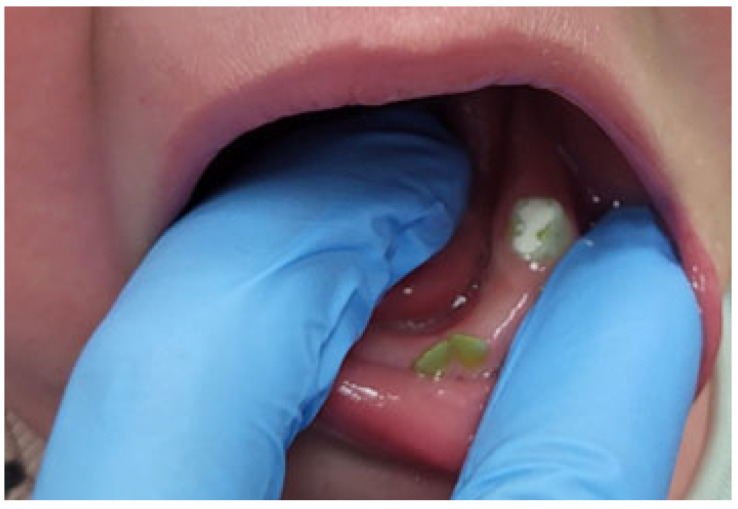
Intristic green discoloration of the mandibular primary teeth with mineralization defects. Teeth 74 and 81 show mesial rotation. Tooth 74 is restored with glass-ionomer cement (Equia Forte HT (GC, Tokyo, Japan)) on the occlusal surface. The restoration illustrates minimally invasive interim management of enamel breakdown with exposed dentin.

The distinction from early childhood caries was based on the absence of plaque-associated cavitation, excellent oral hygiene, low cariogenic exposure, and the distribution of developmental defects on newly erupted teeth. The defects in teeth 74 and 84 were treated using the atraumatic restorative treatment approach and the defects were restored with glass-ionomer cement (Figure 3). Fluoride varnish containing 5% sodium fluoride was applied to the labial surfaces of teeth 52–62 because of enamel chipping near the incisal edges. The ART protocol was performed under relative isolation with cotton rolls and gauze as tolerated. The dentin was assessed as soft as it deformed when excavator was pressed onto it and was easily scooped up with little force being required. Soft dentin was removed with LM-Dental excavators until firm resistance was felt. Then dentin washed with distillate water, delicately dried using air from a dental syringe. Cotton rolls isolation was used during tooth restoring with a bulk-fill reinforced highly viscous glass-ionomer cement Equia forte HT (GC Corporation, Tokyo, Japan). The capsule with the glass-ionomer material was activated in the mixer for 10 s, according to manufacturer indication. The capsule was loaded into an applicator, and Equia Forte HT was inserted into the cavity in one increment, then packed and contoured. The restoration was protected with petroleum jelly. No conditioning was used, as according to the manufacture information preconditioning of the cavity is an optional step and is not mandatory for sufficient bonding [14].

Material selection was based on the child’s very young age, limited cooperation, fluoride release, chemical adhesion, and the need for minimally invasive interim protection of exposed dentin.

The parents were instructed to continue brushing of their child’s teeth with 1000 ppm fluoride toothpaste in a rice-grain amount and applying of casein phosphopeptide-amorphous calcium phosphate (CCP-ACP) cream directly on the teeth after toothbrushing, before sleeping. A control visit was scheduled after two months.

### 3.6. Third Dental Visit—20 Months Chronological Age, 16.5 Months Corrected Age

During the third visit, the child was 20 months old chronologically and 16.5 months old according to corrected age. Cooperation was again limited because of age.

**Figure 4:**
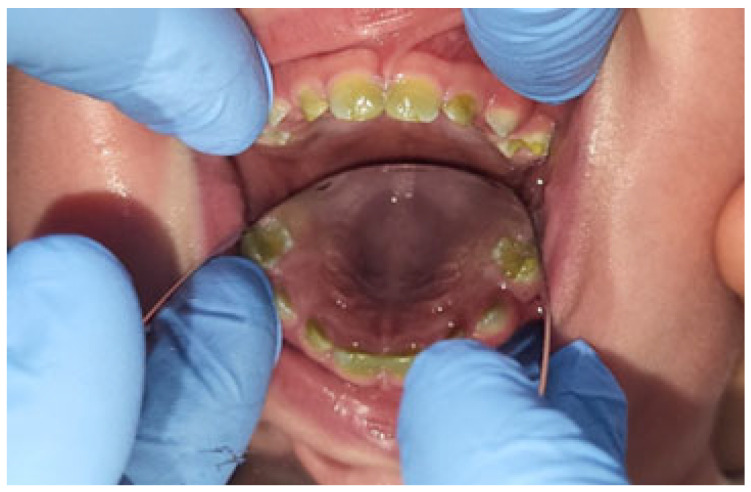
Green discoloration of the entire crowns of maxillary primary teeth in an extremely preterm infant with a history of neonatal cholestasis. Loss of enamel on the labial surfaces of incisors and occlusal surfaces of the first primary molars in maxilla. Partially erupted canines are affected to lesser degree—green staining is reduced to only cuspal region.

The following primary teeth were present: 54, 53, 52, 51, 61, 62, 63, 64, 74, 73, 72, 71, 81, 82, 83, and 84. Teeth 71 and 81 remained in mesial rotation. Teeth 72, 73, 82, and 83 were partially erupted to approximately one-third of crown height (Figure 4).

All newly erupted teeth showed light green discoloration. Tooth 64 showed absence of enamel on the occlusal surface, with a depression involving the entire occlusal surface. The tooth was green in color, and the exposed dentin was soft. The defect was prepared with an excavator and restored with glass-ionomer cement, as described above. A subsequent visit was scheduled after seven days for treatment of tooth 54.

Restorations in teeth 74 and 84 showed satisfactory retention and good marginal integrity.

### 3.7. Fourth Dental Visit—20 Months Chronological Age, 16.5 Months Corrected Age

During the fourth visit, performed seven days after the previous appointment, the child was still 20 months old chronologically and 16.5 months old according to corrected age. The same primary teeth were present: 54, 53, 52, 51, 61, 62, 63, 64, 74, 73, 72, 71, 81, 82, 83, and 84.

The teeth showed generalized light green discoloration, thin enamel, and brittle enamel structure. Tooth 54 presented with absence of enamel and a depression involving the entire occlusal surface. The exposed dentin was green in color and soft. Treatment was performed with an excavator, and the defect was restored using glass-ionomer filling. Restorations of the other first molar teeth were present and satisfactory according to criteria for ART restoration (good marginal adaptation satisfactory integrity, no fracture of the restoration) [15]. The parents were informed that if progressive enamel fractures, attrition, or structural breakdown occurred, the preferred restorative option for affected primary molars would be full coronal coverage with prefabricated stainless steel crowns. At this stage, the parents did not give consent to the placement of stainless steel crowns.

The parents were instructed to continue non-invasive preventive care, including casein phosphopeptide-amorphous calcium phosphate cream and twice-daily brushing with 1000 ppm fluoride toothpaste in a rice-grain amount. The child remained under regular care of the Pediatric Dentistry Outpatient Clinic. The next follow-up visit was scheduled after two months.

### 3.8. Diagnosis

Based on clinical findings and neonatal history, the final clinical diagnosis was presumptive bilirubin-related chlorodontia of the primary dentition coexisting with hypomineralisation and post-eruptive enamel breakdown in an extremely preterm infant. The child’s mother reported noticing enamel chipping off in the incisal region of the upper deciduous incisors, and the enamel edges were found to be sharp upon examination with a probe, which may confirm post-eruptive enamel breakdown. In the case of the first molar teeth, the enamel edges in the occlusal area with exposed dentin were also irregular. However, the coexistence of this with the enamel hypoplasia described by the Federation Dentaire Internationale (FDI) as the partial or complete absence of enamel over a considerable area of a tooth crown cannot be excluded [14]. Additionally, a potential cause of enamel loss was impossible to determine. The diagnosis of bilirubin-related staining was considered clinically probable due to the temporal and biological association between severe neonatal hyperbilirubinemia, and green intrinsic discoloration of the primary teeth [1,2,3,4].

Histological analysis that could confirm this diagnosis was not performed because neither was extraction of affected teeth clinically indicated, nor did exfoliation occur during the follow-up period. Therefore, the diagnosis remains clinically probable rather than definitive.

### 3.9. Further Treatment and Follow-Up Plan

- Professional use of topical fluoride for prevention of caries and hypomineralization-related complications.
- Non-fluoride preventive support (CCP-ACP), including remineralization-oriented home care as clinically indicated.
- Restoration of primary molars with prefabricated stainless steel crowns if progressive attrition, enamel fracture, or structural breakdown occurs.
- Possible future esthetic treatment using direct resin composite restorations or prefabricated zirconia crowns for anterior teeth
- Elimination of harmful oral habits, including gradual discontinuation of pacifier use and bottle-feeding patterns that may affect occlusal development.
- Encouragement of age-appropriate chewing of harder foods under safe pediatric dietary guidance.
- Orthodontic consultation around the age of 3 years.
- Long-term dental follow-up, control visits every 3 months, or more often if the parents observe any concerning oral symptoms.

## 4. Discussion

Green intrinsic discoloration of primary teeth is a rare clinical condition that requires a thorough etiological assessment regardless of the patient’s underlying clinical condition. In the presented case, intrinsic green discoloration affected all erupted groups of primary teeth. According to the data from the literature, green pigmentation is generally seen when bilirubin levels are >30 mg/dL [2]. The patient described was an extremely preterm female infant who was exposed to total bilirubin levels up to 30 mg/dL due to cholestasis starting on day 30 of life, with the highest level of bilirubin on her 40th day of life. It suggests presumptive bilirubin-associated chlorodontia, and corresponds with other case reports linking green pigmentation of primary teeth to neonatal hyperbilirubinemia [1,2,3,16,17,18,19] and cholestatic jaundice [4,20,21,22,23,24,25,26,27,28,29,30,31].

When there is an increased level of bilirubin while the teeth’s crowns are formed, it may be incorporated into mineralizing dental tissues and subsequently oxidized to biliverdin [17,21,22]. The gradation of green discoloration and the group of teeth stained are dependent on the period when hyperbilirubinemia occurs during teeth mineralization [22]. Also the intensity of staining is directly related to the duration and severity of the associated underlying disease [21,22]. Green intrinsic pigmentation are mainly reported in primary teeth; the calcification of which starts between 4 and 6 month in utero and continues until 11 months after birth [20,21]. The formation of incisors’ crowns is completed at 1 month after birth and molars and canines by the 6th month after birth [2]. In the case presented, the highest level of bilirubin due to cholestasis jaundice was observed on the 40th day of life; however, the corrected age due to premature birth should be taken into consideration. It seems to explain the most intensive staining of incisors and occlusal one-third of first molars and changes limited to incisal tips of canines, which would have completed after birth [2]. However, the lack of detailed data on duration of hyperbilirubinemia in our patients does not provide objective evidence, demonstrating that the enamel defects are directly related to bilirubin deposition rather than other perinatal or postnatal factors. Permanent dentition is seldom affected, mainly in patients with history of biliary atresia [21]. In soft tissues, bilirubin deposits are also observed during hyperbilirubinemia but they disappear with cell turnover, with the return of normal concentration of blood bilirubin [21,22]. In contrast, after maturation, enamel and dentin do not demonstrate relevant metabolic turnover; consequently, pigments may remain entrapped and discoloration is produced by bilirubin oxidation to biliverdin, which is visible during dental examination of a patient [1]. As a result green discoloration of dentition may be one of the delayed markers of severe neonatal systemic disturbances [1,2,3].

Data from the literature is inconclusive as to whether the pigment deposits are located in both dentine and enamel [1]. According to Naudi et al. [20] the pigment is deposited only in dentine. Additionally, histological analyses carried out by Amaral et al. [18] and Carrillo et al. [16] showed that bilirubin deposits manifest as green-pigmented lines parallel to incremental lines in dentin, but enamel does not exhibit pigmentation. According to Carrillo et al. [16] this can be explained by the fact that odontoblasts synthesize and secrete the organic collagen-rich dentin matrix that subsequently mineralizes, so hyperbilirubinemia at this stage leads to pigment deposition. As a consequence dentin changes its color permanently as this tissue loses metabolic activity after maturation. In contrast, ameloblasts in secretory phase produce a poorly mineralized matrix and then degrade almost all the organic matrix to allow for the increase in mineral content in the enamel during the maturation phase of amelogenesis. Therefore, the green pigment deposited into enamel during the secretory phase of amelogenesis is likely removed during the maturation phase. The visual change in the color of the enamel is caused by the discolored dentin showing through it [11].

An in vitro model for bilirubin deposition in teeth established by stem cells of human exfoliated deciduous teeth (SHED) cultured with bilirubin proved that the pigment is incorporated in hard tissue during tooth germ development, and that bilirubin suppresses cell proliferation and promotes cell death of SHED. Furthermore, odontogenic capacity of SHED was inhibited with the suppression of AKT and extracellular signal-regulated kinase 1 and 2 (ERK1/2) signaling pathways, and the enhancement of nuclear factor kappa B (NF-κB) signaling pathway. It also affected dentin formation [22].

A very notable feature of this case is that discoloration coexisted with apparent developmental defects of enamel, irregular and rough incisal edges, post-eruptive enamel breakdown, and occlusal enamel loss in primary molars, exposing dentin tissue with a soft outer layer. From the clinical approach, this is especially important since the structural disorder may predispose to hypersensitivity, rapid tissue loss, restorative failure, and caries development. The esthetic appearance of teeth must also be taken into consideration. Description of “soft dentin” refers to clinical visual–tactile findings during routine dental examination and should not be interpreted as objective material testing. The patient was born at 25 weeks of gestation, which is classified and categorized as extremely preterm (younger than 28 weeks) with a very low birth weight (910 g) [23]. Additionally, she experienced multiple severe neonatal complications, including cholestasis, necrotizing enterocolitis requiring surgical treatment, bacterial sepsis, bronchopulmonary dysplasia, transient endocrine/metabolic abnormalities, and prolonged intensive care. Exposure to those factors can be detrimental to the processes of tooth bud formation and subsequent tooth development, which extend over a prolonged timeframe. These exposures overlapped with critical windows of enamel secretion and maturation. Systematic reviews and meta-analyses consistently demonstrate that preterm birth and low birth weight are associated with a higher prevalence of developmental defects of enamel in the primary dentition [7,8]. Clinical studies in preterm children also support this association [9,23]. Furthermore, broader evidence on acquired enamel defects suggests a multifactorial pathogenesis involving among other factors: inflammatory burden and medical interventions during early life development [20,22]. Factors associated with extreme prematurity—including very low gestational age and birth weight, low Apgar scores, prolonged hospitalization, and severe neonatal morbidities such as bronchopulmonary dysplasia and necrotizing enterocolitis—may increase both the prevalence and severity of enamel defects [10]. Also, Wagner et al.’s [24] study proved that preterm children with low birth weight, and those with hospitalization in the first year of life, showed an increased risk of DDE in milk dentition. In the case presented, several risk factors occurred simultaneously: high levels of bilirubin, extreme prematurity and the overall burden of systemic complications. All these factors together could have contributed not only to tooth discoloration, but also to mineralization disorders.

Data in the literature on the impact of the use of antibiotics on hypomineralization are inconclusive. A systematic review with meta-analysis on prenatal, perinatal and postnatal events associated with hypomineralized second primary molars (HSPM), conducted by Lima et al. [25], showed that not only low birth weight (OR = 1.50; 95%CI: 1.15–1.96), prematurity (OR = 1.93; 95%CI: 1.37–2.71) and need for an incubator (OR = 1.65; 95%CI: 1.01–2.70), but also the use of antibiotics by the child (OR = 1.24; 95%CI: 1.04–1.48) and fever (OR = 1.37; 95%CI: 1.10–1.72), were the postnatal factors associated with HSPM. It was also proved that MIH (molar incisor hypomineralization) was more common among those children who had taken, during the first year of life, amoxicillin (OR = 2.06; 95% CI, 1.01–4.17) or erythromycin (OR = 4.14; 95% CI, 1.05–16.4), compared with children who had not received treatment [26]. The newest systematic review, with meta-analysis of experimental animal studies, suggest that several antibiotics, including amoxicillin, ampicillin, gentamicin and the macrolides, detrimentally affect ameloblasts capacity for enamel formation resulting in quantitative defects. However, overall, impact of antibiotic administration was heterogeneous, with inconsistency across studies. Reduced enamel thickness associated with amoxicillin exposure (Cohen’s d = 0.79, 95% CI: 0.40–1.36) with a trend toward larger effect sizes at higher experimental doses was observed. Additionally, nonsteroidal anti-inflammatory drugs, including ibuprofen, by COX-2 inhibition appear to lead to a quantitative reduction in calcium and phosphorus in developing enamel [27].

However, Lima et al. suggest that association between the use of antibiotics and hypomineralisation may be due to the infection that motivated antibiotic intake and not directly due to its use. Additionally, high temperature may have interfered with mineralization and altered the expression of some genes essential for normal enamel formation [25]. Another factor that can affect enamel formation is hypoxia, which could have also been experienced by the presented patient due to extreme prematurity and bronchopulmonary dysplasia. An in vitro study proved that hypoxic exposure may disrupt the controlled fine-tuned expression of enamel genes and increase the secretion of pro-inflammatory factors [28]. What is critical, particularly in the case presented, is that primary dentition development begins prenatally. However, in infants born preterm at a very low gestational age, a part of enamel mineralization that would take place in utero might proceed after birth. For example, infants born before the 29th week will miss an important period of tooth development during the third trimester of gestation [22]. Systemic factors that disturb the ameloblasts during the secretory phase cause restriction of crystal elongation and result quantitative defects, such as hypoplasia, whereas disturbances during maturation may lead to qualitative hypomineralization [23]. Rythen et al. [29] proved that the enamel of preterm children, compared to full-term children, had a higher carbon (C) but lower calcium (Ca) content, and lower ratio of calcium/phosphorus (Ca/P), as well as a lower ratio of Ca/C indicating increased enamel porosity. In dentin, the relative values for P were higher, and Ca/P ratio was lower, at the dentin-pulp junction. In the presented case report, the clinical appearance of dentition suggests that both quantitative and qualitative components may have been present, supporting the concept of prolonged or multifactorial impairment. Also, Seremidi et al. [1] observed a coexistence of chlorodontia and enamel hypoplasia of primary incisors canines linked to cholestasis in a prematurely born child. The differential diagnosis of teeth discoloration includes extrinsic staining caused by chromogenic bacteria, dietary pigmentation, internal medication-related discoloration, discoloration related to bilirubin deposition, congenital porphyria, inherited enamel disorders, early childhood caries or isolated developmental enamel defects [21].

Green intrinsic teeth pigmentation is mainly associated with increased levels of bilirubin but must be differentially diagnosed from other intrinsic stains caused by medication, such as tetracycline. In the case reported, extrinsic causes were unlikely since the discoloration involved the entire clinical crowns could have not been removed and co-occurred with abnormal enamel morphology. Excellent oral hygiene reported during follow-up and a non-cariogenic dietary pattern further reduced the likelihood of hygiene or diet-driven etiologies of the defects. The absence of relevant medication exposure associated with intrinsic discoloration also supported a clinically probable bilirubin-related explanation. While inherited enamel disorders can present with generalized structural abnormalities, the clinical history of severe neonatal hyperbilirubinemia with cholestasis, together with prematurity and multisystem complications, could have supported a bilirubin-related discoloration etiology but did not prove causality.

A more detailed differential diagnostic assessment was also considered. Amelogenesis imperfecta was regarded as less likely because the presentation emerged in the context of severe neonatal systemic morbidity, and there was no reported family history or syndromic pattern suggestive of a hereditary, generalized enamel disorder [30]. Localized enamel hypoplasia was also unlikely to account for the generalized discoloration and multisite involvement. Hypomineralized primary second molars were not applicable to the teeth erupted at the reported visits; however, distinction from other hypomineralization phenotypes supports the need for longitudinal follow-up [31]. Medication-induced discoloration, including tetracycline-related staining, was considered unlikely because no relevant exposure was identified in the available medical history. Congenital porphyria and other genetic disorders affecting enamel development were considered less likely in the absence of a compatible systemic history, family history, or typical clinical features; however, genetic or metabolic etiologies cannot be definitively excluded without targeted testing.

A practical diagnostic challenge concerns distinguishing early childhood caries from post-eruptive breakdown of developmentally defective enamel. Occlusal enamel loss and exposed softened dentin tissue were observed in the primary molars in the presented case. However, the clinical context did not support caries as the initiating process. The clinical picture suggested the structural failure of hypomineralized or hypoplastic enamel shortly after eruption. Nevertheless, developmental enamel defects increase susceptibility to primary and secondary caries development as they promote plaque retention and reduce structural resistance. Meta-analytic evidence demonstrates a significant association between developmental defects of enamel and dental caries in the primary dentition particularly among young children [32,33]. Accordingly, even if initial tissue loss is primarily structural, the patient should be considered as high-risk for future caries occurrence and restoration failure. Therefore, preventive management should be intensive and systematic. Regular professional topical fluoride applications, twice-daily toothbrushing with age-appropriate fluoride concentration toothpaste, dietary counseling, and short recall intervals are highly recommended. Parental engagement resulting in good oral hygiene observed in this case is an important protective factor. However, the underlying structural vulnerability still warrants long-term monitoring and follow-up.

In this case, the absence of visible plaque accumulation, the recorded OHI = 0 and API = 0, the low-cariogenic diet, and the rapid breakdown of newly erupted teeth with visibly defective enamel favored developmental post-eruptive enamel breakdown over early childhood caries as the initiating process. However, because softened exposed dentin was present and developmental enamel defects increase caries susceptibility, the child was managed as high-caries-risk and placed under intensive preventive follow-up. Atraumatic restorative treatment and interim therapeutic restoration principles support minimally invasive approaches in young children or those with limited ability to cooperate [32].

In the case presented, glass-ionomer restorative materials with fluoride release provided interim protection of exposed dentin, reduced the risk of sensitivity, and helped maintain function. The use of glass-ionomer cement can be rationalized by its properties such as biocompatibility, chemical adhesion to enamel and dentin and remineralization ability due to fluoride release [34]. Additionally, the child’s behavior in the presented case limited the possibility of introducing a proper adhesive procedure for composite resin. Equia Forte HT was chosen as the restorative material as the manufacturer claims that it is dedicated to hypomineralized molar teeth. Furthermore, it was shown that the survival rate of this glass hybrid was satisfactory for up to two to three years, even in first molars affected by severe molar incisor hypomineralization (MIH) [35]. At the same time, clinicians should recognize that intracoronal restorations may have limited longevity when enamel is thin, brittle, or susceptible to marginal breakdown. If enamel breakdown progresses, full coronal coverage with preformed stainless steel crowns should be reconsidered for affected primary molars. Evidence and clinical recommendations support their effectiveness and longevity in primary teeth with extensive tissue loss, developmental defects, and high risk of restoration failure [36,37]. Because the observation period after restoration was relatively short, these findings document early clinical retention only and cannot be interpreted as evidence of long-term restorative effectiveness.

The eruption pattern and tooth-positioning abnormalities observed in this case also warrant longitudinal assessment. Because corrected age is essential when evaluating extremely preterm infants, premature labeling of delayed eruption should be avoided. Nevertheless, the combination of prematurity, systemic disease, abnormal mineralization, and eruption irregularities justifies periodic monitoring of dental development and, when indicated, referral for orthodontic consultation.

This case contributes to the limited literature by suggesting that presumptive bilirubin-associated chlorodontia can coexist with severe structural enamel defects and clinically significant post-eruptive breakdown, necessitating active prevention and restorative care. It also emphasizes that green discoloration should not be dismissed as a purely esthetic finding; in medically complex, extremely preterm children, it may reflect substantial neonatal systemic disease and may coincide with enamel fragility carrying long-term oral health consequences.

### Limitations

This case report has several important limitations.

First, histological analysis, SEM analysis or Micro-CT and The Energy Dispersive X-ray (EDX) microanalysis evaluation of the samples was not possible due to the child’s age at the time of admitting and during follow-up period. No tooth needed to be extracted, and neither did exfoliation occur during this period. Moreover, extracting the teeth for performing the above tests would be highly unethical because they would not be clinically justified. In addition, no Spectrophotometric color analysis or Quantitative color measurements were carried out.

Second, precise neonatal data on bilirubin kinetics were unavailable in the records reviewed for this report. Although the reported peak total bilirubin level approached 30 mg/dL, the exact peak value, duration of hyperbilirubinemia, bilirubin fractionation, and the timing of bilirubin exposure relative to tooth mineralization could not be established. This limits causal inference.

The records also did not provide the duration of cholestasis, detailed phototherapy parameters, or a complete chronological profile of NICU exposures and medication use during the period relevant to tooth development.

Third, this case cannot disentangle the potential contribution of bilirubin exposure from other prematurity-related factors that may have influenced the enamel defects. The presence of cholestasis, necrotizing enterocolitis and sepsis-related inflammatory burden, bronchopulmonary dysplasia, metabolic instability, nutritional disturbances, prolonged intensive care exposure, and multiple concomitant medications represents substantial confounding. Liver-function tests and detailed metabolic parameters were also not available in sufficient chronological detail to correlate them precisely with odontogenesis. Accordingly, the enamel defects are best interpreted as multifactorial rather than attributable to bilirubin exposure alone.

Fourth, the follow-up period in this case remains relatively short, and the diagnosis of developmental enamel defects was based on clinical findings and radiographic assessment was not performed due to the patient’s age and limited cooperation. These factors limit the precision of defect classification and longitudinal assessment. Consequently, defect severity could not be objectively quantified. In addition, photographic documentation was not acquired under a fully standardized imaging protocol, which limits objective color comparison between visits.

Fifth, objective color measurements were not performed; teeth color was assessed clinically and descriptively during intraoral examination. Clinical photographs were obtained as routine documentation during follow-up visits; camera settings, illumination, and color calibration were not standardized.

## 5. Conclusions

This case highlights that bilirubin-related discoloration should be included in the differential diagnosis of intrinsic green discoloration of primary teeth, particularly in extremely preterm children with a history of neonatal jaundice, cholestasis, or severe systemic illness. In such children green discoloration may coexist with enamel fragility and post-eruptive breakdown; therefore, management should extend beyond reassurance or esthetic.

Early recognition of the defect, parental counseling, intensive preventive care, and minimally invasive restorative treatment when needed are essential. In the presence of the progressive structural breakdown of primary molars, full coronal coverage with prefabricated stainless steel crowns should be considered. Long-term follow-up is required to monitor the eruption timing, enamel integrity, restorations survival, caries risk, development of occlusion, and potential future esthetic needs. Because this is a single case report, these observations are not generalizable and should be regarded as clinically informative rather than as definitive evidence of causality. The clinical message is therefore limited to recognition, differential diagnosis, and follow-up planning in comparable high-risk patients, not to generalize estimations of prognosis or causal risk.

## Figures and Tables

**Table 1:** Medications used in the patient’s neonatal therapy, mentioned in the Patient Discharge Forms.

International Nonproprietary Name/Active Substance(s)	Substance Category
Ampicillin	beta-lactam antibiotic; aminopenicillin
Meropenem	carbapenem antibiotic
Vancomycin	glycopeptide antibiotic
Metronidazole	nitroimidazole antibacterial and antiprotozoal agent
Sulfamethoxazole + Trimethoprim	combination antibacterial agent
Amikacin	aminoglycoside antibiotic
Ceftazidime	third-generation cephalosporin antibiotic
Fluconazole	triazole antifungal agent
*Lactobacillus rhamnosus* R0011 + *Lactobacillus helveticus* R0052	probiotic preparation
Poractant alfa	pulmonary surfactant
Ibuprofen	non-steroidal anti-inflammatory drug
Omeprazole	proton pump inhibitor
Ursodeoxycholic acid	bile acid; choleretic/hepatobiliary agent
Pentoxifylline	methylxanthine derivative; hemorheologic agent
Human insulin	peptide hormone; antidiabetic agent
Red blood cell transfusion	supportive treatment
Paracetamol	analgesic and antipyretic
Epinephrine	vasopressor
Dopamine	catecholamine inotrope/vasopressor
Hydrocortisone	glucocorticoid
Fentanyl	opioid analgesic
Midazolam	benzodiazepine sedative
Levetiracetam	antiepileptic agent
Phenobarbital	barbiturate antiepileptic/sedative
Ketamine	dissociative anesthetic
Heparin	anticoagulant
Furosemide	loop diuretic
Antithrombin III/antithrombin alfa	anticoagulant replacement; serine protease inhibitor
Ipratropium bromide + fenoterol hydrobromide	bronchodilator combination; anticholinergic + beta2-agonist
Budesonide	corticosteroid; inhaled glucocorticoid
Cholecalciferol	vitamin D3 preparation
Retinol	vitamin A preparation
Alpha-tocopherol	vitamin E preparation
Human varicella-zoster immunoglobulin	specific immunoglobulin; passive immunization

**Table 2:** Clinical timeline of neonatal history, dental findings, and management. Because exact bilirubin kinetics and detailed exposure chronology were unavailable, the timeline correlates neonatal events, broad odontogenesis-related windows, eruption chronology, and dental management descriptively rather than quantitatively.

Age/Period	Clinical Findings	Management
Birth/neonatal period	Born at 25 weeks of gestation, birth weight 910 g, recurrent hyperbilirubinemia reported between the second and fifth days of life, with total bilirubin levels up to approximately 30 mg/dL, direct bilirubin: 19 mg/dL, exact duration, and fractionation is unavailable, cholestasis, NEC, sepsis, BPD, ROP. Duration of cholestasis, detailed phototherapy parameters, bilirubin fractionation, and exact exposure chronology were unavailable.	NICU care, phototherapy, surgery for NEC, systemic treatment. Detailed phototherapy parameters unavailable.
15 months chronological/ 11.5 months corrected	Teeth 52, 51, 61, 62, 71, 81 erupted; generalized green discoloration; rough incisal edges; teeth 71 and 81 erupting in mesial rotation.	5% NaF varnish, fluoride toothpaste 1000 ppm, CPP-ACP cream, follow-up.
18 months chronological/ 14.5 months corrected	Teeth 74 and 84 erupted; less intense green discoloration in molars; enamel loss and soft dentin on occlusal surfaces; OHI = 0, API = 0.	ART with excavator, glass-ionomer restorations in 74 and 84, 5% NaF varnish.
20 months chronological/ 16.5 months corrected	Newly erupted primary maxillary first molars, light green discoloration; brittle enamel; tooth 64 with occlusal enamel loss and soft dentin.	ART and glass-ionomer restoration of 64.
20 months chronological/ 16.5 months corrected	Tooth 54 with occlusal enamel loss, green discoloration, and soft dentin.	ART and glass-ionomer restoration of 54; stainless steel crowns discussed but declined at this stage.

## Data Availability

Data sharing is not applicable to this article because it presents a single anonymized clinical case report and no datasets were generated or analyzed.

## Abbreviations

The following abbreviations are used in this manuscript:

API	Approximal Plaque Index
ART	Atraumatic Restorative Treatment
BPD	Bronchopulmonary Dysplasia
CARE	Case Report Guidelines
CPP-ACP	Casein Phosphopeptide-Amorphous Calcium Phosphate
DDE	Developmental Defects of Enamel
FDI	Fédération Dentaire Internationale tooth notation system
NaF	Sodium Fluoride
NEC	Necrotizing Enterocolitis
NICU	Neonatal Intensive Care Unit
OHI	Oral Hygiene Index
ppm	Parts per million
ROP	Retinopathy of Prematurity
